# Heart-Specific and Conditional Deletion of the *Immt* Gene Reveals Its Role in Regulating Mitochondrial Structure and Total Heart Metabolism

**DOI:** 10.3390/cells15060505

**Published:** 2026-03-12

**Authors:** Yasuhide Kuwabara, Caitlin Keezer, Suh-Chin J. Lin, Akanksha Rajput, Jeffery D. Molkentin

**Affiliations:** 1 Division of Molecular Cardiovascular Biology, Heart Institute, Cincinnati Children’s Hospital Medical Center, Cincinnati, OH 45229, USA; 2 Department of Pediatrics, University of Cincinnati College of Medicine, Cincinnati, OH 45229, USA

**Keywords:** mitochondria, cardiac hypertrophy, cardiomyocyte, metabolism, mitophagy

## Abstract

**Highlights:**

**What are the main findings?**

- The *Immt* gene (Mic60) is required in the mouse heart to maintain mitochondrial structure and function.
- Loss of mitochondria in the heart is eventually lethal, although compensation and temporary metabolic function are preserved by a unique mitochondrial stress response.

**What are the implications of the main findings?**

- Inducible loss of Mic60 in the heart can be used as a model to investigate mitochondrial renewal and biogenic programs in vivo.
- Inducible loss of Mic60 in the heart can also suggest novel metabolic pathways that are induced in the heart to compensate for the loss of mitochondrial oxidative function.

**Abstract:**

Mitochondria comprise ~1/3rd of the volume of an adult ventricular cardiomyocyte. The gene *Immt* encodes the Mic60/Mitofilin protein that is hypothesized to organize the mitochondrial contact site and cristae organization system (MICOS) complex that generates mitochondrial cristae junctions between the inner and outer membranes. To investigate the function of the *Immt* gene in the mouse heart, we generated and characterized mice in which this gene was specifically deleted in the mouse heart using a loxP-targeted allele (*Immt^fl^*^/*fl*^) and either the constitutive heart-specific Myh6-Cre transgene or the conditional Myh6-MerCreMer transgene, each of which showed lethality in several weeks. Hearts from these mice showed progressive hypertrophic cardiomyopathy and failure with lost contractility and lung edema. At the ultrastructural level, hearts from these mice showed extreme abnormalities in mitochondrial architecture characterized by lost cristae junctions, stacking of the inner mitochondrial membranes, mitophagy and areas with complete absence of mitochondria. Analysis of mitochondria showed loss of the MICOS complex of proteins as well as loss of mitochondrial membrane potential (Δψ) and increased expression of mitophagy proteins and mitochondrial biogenesis transcription factors. Hearts from these mice also showed widespread cardiomyocyte necrosis and induction of the universal mitochondrial stress response at the mRNA level, as well as major alterations in cardiac metabolites, suggesting greater use of glucose, ketones and amino acids. We conclude that the *Immt* gene is required for cardiac mitochondrial structure and function, although the ensuing mitochondrial stress response provides molecular clues as to how the heart can compensate metabolically and maintain viability for weeks after mitochondria are absent or unfunctional.

## 1. Introduction

The human heart consumes 6 kg of ATP each day to drive contractility and ion gradients that are required to pump blood [1]. Nearly all of this high-energy phosphate needed each day is generated by mitochondria in the process known as oxidative phosphorylation and the generation of an electrical–chemical gradient. Mitochondria contain two membranes, the outer mitochondrial membrane (OMM) that partitions the organelle from the rest of the cytoplasm and the inner mitochondrial membrane (IMM) that contains the enzymatic complexes whereby substrate utilization generates this electrical–chemical gradient that the ATP synthase (F_1_F_O_-ATPase) uses to generate ATP [1,2]. In the heart, the IMM is highly elaborated and stacked to amplify the effect of this electrochemical gradient for a given amount of mitochondrial area [3]. These stacks of IMM contract the OMM at the cristae junctions (CJ) that are organized by the MICOS complex, comprising Mic60 (mitofilin), Mic10, Mic19, Mic25, Mic13, Mic23/26, Mic27 and possibly Mic14 [3,4], and these proteins are conserved from yeast to vertebrates. This IMM MICOS complex interacts with proteins in the OMM by binding to the translocase of the outer mitochondrial membrane (TOM) family and the sorting and assembly machinery (SAM) family, with representatives being Tom40 and Sam50 [3,4,5]. This MICOS supercomplex is also thought to help organize the enzymes involved in the electron transport chain, the mitochondrial uniporter (MCU) complex and adenine nucleotide translocator (ANT) and phosphate carrier (PIC) for efficient function in generating ATP [6]. The MICOS complex might also be critical in serving as an anchor point with the voltage-dependent anion channel (VDAC) in the OMM for attachment to structures such as the sarcoplasmic reticulum (SR) or attachment to microtubules or intermediate filaments in positioning these organelles within tissues [7,8,9]. Indeed, desmin is thought to directly bind to mitochondria through Mic60 in positioning these organelles within a highly ordered array deep within the sarcomeres of heart and skeletal muscle to provide local ATP [8,9].

In vertebrates, loss of Mic19 or Mic25 results in disassembly of the MICOS complex, similar to loss of Mic60 with siRNA approaches that resulted in defective cristae architecture and loss of the MICOS complex [10,11,12,13]. More recently, mice with global but temporally inducible deletion of the *Immt* gene (Mic60 protein) using a *Rosa26*-CreERT2 approach were shown to be lethal within 12 days of tamoxifen challenge, resulting in severe alterations in mitochondrial architecture and widespread tissue abnormalities [14]. These results suggest that Mic60 and the MICOS complex are critical to mitochondrial function in vivo, although another parallel question we sought to address is the metabolic importance of Mic60 and mitochondria in general in maintaining cardiac function in vivo. For example, mice with heart-specific deletion of genes necessary for mitochondrial function, such as *Tfam* [15], *Twnk* [16,17], *Lrpprc* [18], *Polrmt* [19], or *Mterf4* [20], resulted in lethality when using a constitutive cardiac-specific Cre expressing transgene, likely due to complete loss of mitochondrial oxidative phosphorylation (OXPHOS) capacity [21]. Here, we deleted the *Immt* gene from the heart using two approaches, each of which caused lethality in mice and a near-complete loss of mitochondrial function and integrity. Remarkably, these mice lived up to 10–12 weeks of age despite the loss of mitochondrial function by inducing a compensatory mitochondrial stress response gene signature that suggests ongoing mitochondrial biogenesis with mitophagy, as well as the induction of metabolic shunt pathways that allows greater use of glucose and other substrates.

## 2. Materials and Methods

### 2.1. Animals

The conditional *Immt* (Mic60) mouse line was created from an embryonic stem (ES) cell clone HEPD0570_5_G06 using a knock-out first allele (*Immt^tm1a(EUCOMM)Hmgu^*), purchased from European Mouse Mutant Cell Repository (EuMMCR). ES cell aggregation with 8-cell embryos was used to generate chimeric mice for this *Immt* locus. Germline transmitting male chimeras were crossed with *Rosa26-FLPe* females (#009086, B6.129S4-*Gt(ROSA)26Sor^tm1(FLP1)Dym^*/RainJ, the Jackson Laboratory, Bar Harbor, ME, USA) to remove the neomycin cassette and to generate a conditional allele with loxP sites flanking exon 4 of the *Immt* locus. Transgenic mice with the α-myosin heavy chain (Myh6) promoter directing Cre recombinase (Myh6-Cre Tg) or an inducible Myh6-MerCreMer line in the C57BL/6 background were described previously [22]. Both transgenic lines are also at the Jackson Laboratory (#009074: Tg(Myh6-cre)1Jmk/J: Myh6-Cre and #005650: *A1cf^Tg(Myh6-cre^*^/Esr1*)1Jmk^/J: Myh6-MerCreMer). Since the Myh6-Cre Tg line is X-linked, only male mice were used in the experiments involving *Immt* gene-deleted mice. However, both sexes were utilized in Myh6-MerCreMer inducible *Immt* gene-deleted mice. As control mice of *Immt^fl^*^/*fl-Myh6-Cre*^ mice, male *Immt*^+/+*-Myh6-Cre*^ mice were used. *Ppif* gene-targeted mice were also generated previously in the C57BL/6 background [23], also held at the Jackson Laboratory (#009071, B6;129-*Ppif^tm1Jmol^*/J). To ablate the *Immt* locus temporally with the Myh6-MerCreMer line, tamoxifen (T5648, Sigma-Aldrich, St. Louis, MO, USA) was dissolved in corn oil (C8267, Sigma-Aldrich), and the solution was injected intraperitoneally into *Immt^fl^*^/*fl-Mhy6-MCM*^ mice at 6 weeks of age at a dose of 40 mg/kg body weight 3 times every other day. *Immt*^+/+*-Mhy6-MCM*^ mice were used as a control. The individual mouse was considered the experimental unit unless otherwise noted. Sample sizes were determined empirically.

### 2.2. Animal Welfare and Ethics

Animals were handled in accordance with the principles and procedures of the Guide for the Care and Use of Laboratory Animals. All proposed procedures were approved by the Institutional Animal Care and Use Committee (IACUC) at Cincinnati Children’s Hospital Medical Center (CCHMC) in the USA. The IACUC ID is 2024-0043 and the approval date is 4 September 2024. Animal groups and experiments were handled in a blinded manner where possible. There is no excluded mouse during the experiments. Randomization was not performed, given that all mice were of the same genotype and identical strain, and only age-matched littermates were compared. ARRIVE guidelines were followed in all mouse experimentation [24]. No human materials or subjects were used. All mice were housed in a germ-free barrier environment with free access to food and water, with 14 h day/10 h night cycles, and observed every day by veterinary staff. Pain management in mice is discussed below.

### 2.3. Echocardiography

Mice were anesthetized by 3% isoflurane for induction of anesthesia, which was maintained with 1.7% isoflurane during echocardiography. Images were recorded on the ventral side of the chest in a blinded manner. The echocardiography was conducted using Vevo 3100 (Fujifilm Visualsonics, Toronto, ON, Canada). The Vevo images were analyzed by Vevo Lab software (Fujifilm Visualsonics, software version 5.10.0). Parameters, including ejection fraction (EF), were determined using M-mode images. The analyses were also conducted in a blinded manner.

### 2.4. Survival Analysis

Animal care technicians, under the supervision of 2 veterinarians, monitored the mice every day and assessed well-being according to the IACUC-approved guidelines for this study. If mice were deemed excessively moribund, they were first treated by placing gel diet on the bottom of the cage that also hydrates, but if they progressed and were no longer able to acquire nutrients and hydration, the mice were removed from the study and euthanized (this was considered a death event in the survival studies), as approved within the IACUC protocol and guidelines by the Office of Laboratory Animal Welfare.

### 2.5. Western Blot

Tissue homogenization and Western blotting were conducted as described previously [22]. Briefly, tissue samples were homogenized in 10 mM Tris (pH 7.5), 150 mM NaCl, 4% glycerol, 0.5 mM NaMetabisulfite, 1% Triton X-100, 0.05% sodium dodecyl sulfate (SDS), 0.1% NaDeoxycholate with complete mini proteinase inhibitor cocktail (#04693124001, Roche, Basel, Switzerland) and 100× EDTA (500 mM) just before homogenization. After homogenization, the supernatant was centrifuged at 14,000× *g* for 15 min at 4 °C. The supernatant was then snap-frozen by liquid nitrogen and stored in a −80 °C freezer until use.

Protein concentrations of the homogenates were determined by the DC protein assay kit (#5000112, Bio-Rad, Hercules, CA, USA), and then the samples were subjected to Western blotting, prepared by adding 5× SDS loading dye and boiled at 97 °C for 10 min. The samples were subjected to polyacrylamide gel electrophoresis (PAGE). The gels were then transferred to Immobilon-P PVDF membranes (#IPVH00010, Millipore Sigma, Burlington, MA, USA). The membranes were blocked with a 5% milk protein solution in 1× phosphate-buffered saline (PBS) for 1 h and then incubated with primary antibody diluted as indicated below in 5% milk protein/Tris-buffered saline with 0.1% Tween 20 (TBST) at 4 °C overnight. The membranes were then washed with TBST and incubated with secondary indicator antibody (IRDye secondary antibodies, LI-COR, 1:3000) in 5% milk protein/TBST with 0.01% SDS at room temperature for 1 h. After washing with TBST 3 times, the membranes were then scanned by the Odyssey CLx imaging system with Image Studio software version 6.1 (LI-COR, Lincoln, NE, USA).

Primary antibodies used for Western blots were anti-Mitofilin (Mic60 or Immt) (#10179-1-AP, Proteintech, Rosemont, IL, USA, 1:1000), anti-Mic19 (#25625-1-AP, Proteintech, 1:1000), anti-Sam50 (#28679-1-AP, Proteintech, 1:1000), anti-PINK1 (#BC100-494, Novus Biologicals, Centennial, CO, USA, 1:1000), anti-LC3 (#3868, Cell Signaling Technology, Danvers, MA, USA, 1:1000), anti-p62/SQSTM1 (#P0067, Sigma Aldrich, 1:1000), anti-LAMP2 (#ABL-93, Developmental Studies Hybridoma Bank, 1:50), anti-LIMPII (#ab176317, abcam, Cambridge, UK, 1:1000), anti-PGC1α (#ab54481, abcam, 1:1000), anti-PGC1β (#ab176328, abcam 1:1000), anti-MFN2 (#9482, Cell Signaling Technology, 1:1000), anti-MFN1 (#ab104274, abcam, 1:750), anti-Ubiquitin (#sc-8017, Santa Cruz, Dallas, TX, USA, 1:200), anti-GAPDH (#10R-G109A, Fitzgerald, Acton, MA, USA, 1:5000), anti-Mic26 (#MA5-15493, Thermo Fisher Scientific, Waltham, MA, USA, 1:1000), anti-Mic27 (#28514-1-AP, Proteintech, 1:1000), anti-Mic10 (#31561-1-AP, Proteintech, 1:500), anti-SESN2 (#10795-1-AP, Proteintech, 1:1000), anti-cGas (#31659, Cell Signaling Technology, 1:1000), anti-cMYC (#10828-1-AP, Proteintech, 1:2000), anti-GSDMA (#30354-1-AP, Proteintech, 1:2000), anti-OPTN (#10837-1-AP, Proteintech, 1:5000), anti-OPA1 (#ab157457, abcam, 1:1000), and anti-DRP1 (#8570, Cell Signaling Technology, 1:1000).

### 2.6. RNA Isolation, Bulk RNA Sequencing, and Bioinformatic Analysis

Total RNA was isolated with TRIzol reagent (#15596018, Thermo Fisher Scientific) from heart tissue as previously described [25]. RNA quality was assessed by RNA 6000 Nano Assay (#5067-1511, Agilent, Santa Clara, CA, USA). Library preparation and Illumina platform bulk RNA sequencing were carried out by Illumina Inc. (San Diego, CA, USA) using Novaseq 6000 for the 20-week RNA data or Plasmidsaurus, South San Francisco, CA, USA (10 M deduplicated Illumina reads) for the 10-week RNA data. Bioinformatic analysis of the RNA sequencing data was performed to show differentially expressed genes (DEGs) in hearts of *Immt^fl^*^/*fl-Myh6-MCM*^ mice versus *Immt*^+/+*-Myh6-MCM*^ mice at 20 weeks after tamoxifen injection. The RNA-seq data sets are deposited in the Gene Expression Omnibus (GEO) repository as accession number GSE312894. We also examined hearts of *Immt^fl^*^/*fl-Myh6-MCM*^ mice at 10 weeks after tamoxifen injection versus age-matched *Immt*^+/+*-Myh6-Cre*^ mice. The RNA-seq data sets are deposited in the GEO repository as accession number GSE322575. For the *Immt* dataset, a volcano plot was generated using ggplot2 by plotting log_2_fold-change (log_2_FC) against log_10_(adjusted *p*-value), with genes classified as upregulated, downregulated, or not significant according to adjusted *p*-value < 0.05 and |log_2_FC| > 1. Horizontal and vertical dashed lines indicate the adjusted *p*-value and fold-change cutoffs. Gene ontology enrichment analysis for hearts of *Immt^fl^*^/*fl-Myh6-MCM*^ mice was performed on the subset of upregulated genes (log_2_FC > 2, *p*_adj_ < 0.05) using clusterProfiler version 3.15+ and the org.Mm.eg.db annotation package, restricting the analysis to biological process terms and applying Benjamini–Hochberg correction. The top 20 categories were visualized as a custom dot plot showing gene counts and adjusted *p*-values. For the six-model comparison, RNA-seq datasets for cardiac-specific *Tfam*, *Twnk*, *Lrpprc*, *Polrmt*, and *Mterf4* knockout mouse models were obtained from Supplementary File 4 in a previous report [21]. The raw RNA-seq data for these 5 heart-specific gene-deleted mice were also deposited in the GEO repository under accession number GSE96518. Each dataset was processed in R to extract gene symbols. If genes have a Benjamini–Hochberg adjusted *p*-value < 0.05 and log_2_FC > 2, the genes were considered as DEGs. Log_2_FC values of the DEGs overlapping all six knockout mice were assembled into a matrix (*Immt*, Twnk, *Tfam*, *Polrmt*, *Lrpprc*, *Mterf4*) and displayed as a clustered heatmap using ComplexHeatmap with a blue–white–red color scale to represent relative down- and upregulation.

### 2.7. Immunohistochemistry

Immunohistochemistry was conducted as described previously [22]. Briefly, mouse hearts were perfused with relaxing buffer for 5 min, followed by perfusion fixation with 4% paraformaldehyde (PFA) for 5 min. The hearts were excised and immersed in 4% PFA for 5 h at 4 °C. After washing 3 times in 1× PBS, the tissue was embedded in O.C.T. compound (#4583, Tissue-Tek, Torrance, CA, USA). The frozen blocks were cut using a cryostat (#CM1860, Leica, Wetzlar, Germany) at −20 °C to generate 7 µm thick sections. The sections were washed with 1× PBS 3 times, and then incubated in 0.1% Triton X-100/1× PBS for 10 min. Blocking was conducted using 5% goat serum in 1× PBS for 15 min and primary antibodies were added for overnight incubation. The primary antibodies were rabbit anti-Mic19 (#25625-1-AP, Proteintech, 1:200), rat anti-CD45 (#14-0451-82, Thermo Fisher Scientific, 1:250), rat anti-CD68 (#ab53444, abcam, 1:200), and mouse anti-ACTN2 (A7811, Millipore Sigma, 1:300). The next day, the slides were washed with 1× PBS 3 times and secondary antibodies were added with 1:400 dilution. The secondary antibody was Alexa Fluor 568 goat anti-rabbit IgG (#A-11011, Invitrogen, Carlsbad, CA, USA), Alexa Fluor 568 goat anti-rat IgG (#A-11077, Invitrogen), and Alexa Fluor 488 goat anti-mouse IgG1 (#A-21121, Invitrogen). For membrane staining, Alexa Fluor 488-conjugated wheat germ agglutinin (WGA) (#W11261, Thermo Fisher Scientific) was also added together with the secondary antibody. After 1 h incubation at room temperature, the slides were washed with 1× PBS 3 times and mounted using ProLong Diamond antifade mountant with 4′,6-diamidino-2-phenylindole (DAPI) (#P36962, Thermo Fisher Scientific). The images were captured using an A1 confocal microscope with NIS-elements software version 6.10.01 (Nikon, Tokyo, Japan).

### 2.8. Measurement of Cardiomyocyte Cross-Sectional Area

Heart frozen blocks prepared as described above were used for 7 μm thick sections of the heart’s short axis. After Alexa Fluor 488-conjugated WGA (#W11261, Thermo Fisher Scientific) staining, the images of the base of left ventricular papillary muscles were taken and at least 500 cardiomyocyte cross-sectional areas (CSA) were measured. The average was considered as the cardiomyocyte CSA of the heart.

### 2.9. Evans Blue Dye Uptake Experiment In Vivo

Evans blue dye (EBD) (#E2129, Sigma-Aldrich) at 100 mg/kg body weight was injected intraperitoneally and 24 h later, the heart was excised. After washing in ice-cold PBS, the heart was directly embedded in O.C.T. compound (#4583, Tissue-Tek). The frozen blocks were cut using a cryostat (#CM1860, Leica) to generate 7 μm thick sections. For membrane staining, Alexa Fluor 488-conjugated WGA (#W11261, Thermo Fisher Scientific) staining was conducted for 1 h at room temperature. The images were captured using an A1 confocal microscope with NIS-elements software (Nikon). The EBD-positive cardiomyocytes were observed directly by the Texas Red channel.

### 2.10. Mitochondrial DNA Quantification

Mitochondrial DNA (mtDNA) was quantified as described previously [26]. Briefly, DNA was extracted from ~10 mg of heart tissue using a KingFisher Flex benchtop automated extraction instrument (#5400620, Thermo Fisher Scientific). The extracted DNA was diluted to 20 ng/mL and quantitative PCR was conducted on both *Cox1* for the mtDNA and *Actb* for a nuclear DNA (nucDNA) with SsoAdvanced Universal SYBR Green Supermix (#1725274, Bio-Rad) in CFX Opus 96 Real-Time PCR System (#12011319, Bio-Rad). mtDNA/nucDNA was calculated by the 2^−ΔΔCt^ method. Primer sequences used are as follows. mmu-*Cox1* Forward: 5′-TCTGTTCTGATTCTTTGGGCAC-3′ and mmu-*Cox1* Reverse: 5′-AGCCAATAGACATTATTGCTCATAC-3′. mmu-*Actb* Forward: 5′-CTGTCGAGTCGCGTCCACC-3′ and mmu-*Actb* Reverse: 5′-CAGTGAGGTACTAGCCACGAGA-3′.

### 2.11. Transmission Electron Microscope Analysis

Hearts were perfused with relaxation buffer (100 mM KCl, 5% Dextrose, 30 mM 2,3-Butanedione monoxime in 1× PBS) for 5 min, followed by perfusion with fixation buffer (1% PFA, 2% glutaraldehyde in 100 mM sodium cacodylate, pH 7.4) for 5 min. The hearts were then removed and sectioned into 0.5 mm squares and placed in fixation buffer overnight. Post-fixation was conducted in 1% OsO_4_ for 2 h. Ultrathin sections were counterstained with uranyl acetate and lead salts as described previously [22]. Images were obtained using an HT7800 transmission electron microscope (Hitachi, Tokyo, Japan) connected to a digital camera (AMT, Woburn, MA, USA, Biosprint16) in a blinded manner.

### 2.12. TMRE Staining in Adult Cardiomyocyte

Adult cardiomyocytes were enzymatically isolated with Langendorff perfusion [27] and individual cardiomyocytes were plated on µ-Slide 4 well (#80426, ibidi, Gräfelfing, Germany) coated with laminin as detailed previously [28]. After 90 min incubation, the media was changed to 50 nM tetramethylrhodamine ethyl ester (TMRE) (#T669, Thermo Fisher Scientific) in 1× PBS and the cardiomyocytes were incubated for 60 min. Then, the media was replaced with 1× PBS and images were captured using an A1 confocal microscope with NIS-elements software (Nikon).

### 2.13. Metabolomics Analysis

All nuclear magnetic resonance (NMR) spectroscopy and metabolomics analysis were performed at the Translational Metabolomics Facility (RRID: SCR_022636) in Cincinnati Children’s Hospital Medical Center [29]. Briefly, mouse heart tissue was homogenized in PowerBead Tubes with 2.8 μm ceramic beads (#13114-50, Qiagen, Venlo, The Netherlands) at 5000× *g* for 30 s twice at room temperature. Modified Bligh and Dyer extraction was performed to obtain polar metabolites [30,31,32]. NMR samples were prepared in 103.5 mm × 5 mm NMR tubes (Bruker, Billerica, MA, USA) with 600 mL NMR buffer, which is 100 mM phosphate buffer (pH 7.3), 1 mM 3-trimethylsilyl 2,2,3,3-d4 propinoate (TMSP), and 1 mg/mL sodium azide in D_2_O. All NMR spectra were acquired on a Bruker Avance III HD 600 MHz spectrometer with a 5 mm, BBO Prodigy probe and processed with Topspin 3.6 software (Bruker Analytik, Rheinstetten, Germany). Chemical shifts were assigned to metabolites based on 1D ^1^H, 2D TOCSY and HSQC NMR experiments with reference spectra found in databases, Human Metabolome Database (HMDB) [33], and Chenomx NMR Suite profiling software (Chenomx Inc., Edmonton, AB, Canada, version 8.1). The concentrations of the metabolites in polar extracts were calculated using Chenomx software based on the internal standard, TMSP (1 mM). Through this metabolomics analysis, 45 metabolites were quantified in all 6 heart samples, including 3 controls and 3 *Immt* gene-deleted hearts. Metabolite concentrations were normalized to the original tissue weights and utilized for the subsequent data analyses using R Studio version 2026.01.1 and MetaboAnalyst 5.0 [34].

### 2.14. Statistics

For the analysis of two groups, we first assessed whether the data were normally distributed unless otherwise noted. If the data were normally distributed, we performed unpaired two-tailed t-tests. If not, we performed Mann–Whitney (non-parametric) tests. For more than 2 groups, one-way analysis of variance (ANOVA) with Tukey’s post hoc test was performed. In metabolome analysis, we conducted unpaired two-tailed t-tests for metabolites. For survival analysis, the log-rank test was conducted. A *p*-value less than 0.05 was considered statistically significant. All statistical analyses were conducted using GraphPad Prism 10 (GraphPad Software, San Diego, CA, USA) unless otherwise noted.

## 3. Results

### 3.1. Cardiomyopathic Phenotype in Heart-Specific Immt Gene-Deleted Mice

**Figure 1:**
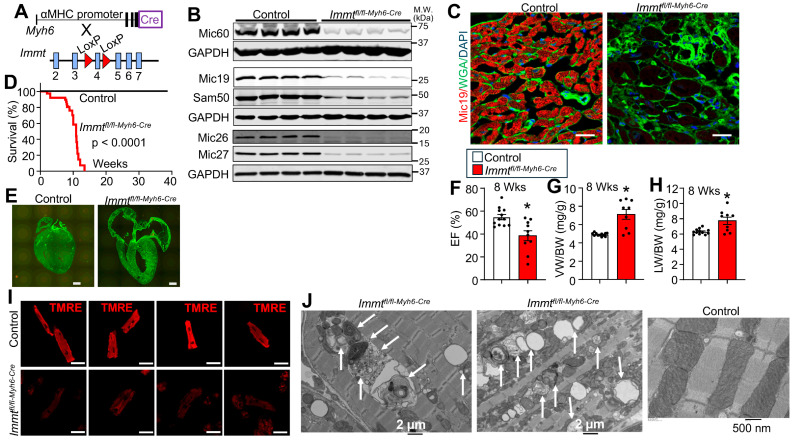
Cardiomyocyte-specific *Immt* gene deletion in mice generates lethal cardiomyopathy. (**A**) To generate cardiomyocyte-specific *Immt* gene-deleted mice (*Immt^fl^*^/*fl-Myh6-Cre*^), loxP site-targeted *Immt* mice were crossed with αMHC promoter (Myh6)-driven Cre transgenic mice. *Immt*^+/+*-Myh6-Cre*^ mice were used as a control. (**B**) Western blots of Mic60, Mic19, Sam50, Mic26, and Mic27 from hearts of *Immt^fl^*^/*fl-Myh6-Cre*^ mice and controls at 8 weeks of age. Glyceraldehyde 3-phosphate dehydrogenase (GAPDH) was run as a control for protein loading and western conditions. Numbers indicate molecular weight (kDa). (**C**) Representative immunohistochemical images of Mic19 (red) and wheat germ agglutinin (WGA) for membrane staining (green) in heart sections from control or *Immt^fl^*^/*fl-Myh6-Cre*^ mice at 8 weeks of age. Scale bar is 10 µm. (**D**) Survival rate of the *Immt^fl^*^/*fl-Myh6-Cre*^ mice versus control after birth. (**E**) Whole heart histological image for the 2 genotypes of mice shown at 8 weeks of age, stained for Alexa Fluor 488-conjugated wheat germ agglutinin (WGA). Scale bar is 1 mm. (**F**) Ejection fraction (EF) by echocardiography at 8 weeks of age; *n* = 12 in control and *n* = 10 in *Immt^fl^*^/*fl-Myh6-Cre*^. *: *p* = 0.002 vs. control. (**G**,**H**) Ventricular weight/body weight ratio (VW/BW) (**G**), and lung weight/body weight ratio (LW/BW) (**H**) at 8 weeks of age; *n* = 12 in control and *n* = 9 in *Immt^fl^*^/*fl-Myh6-Cre*^. *: *p* < 0.005 vs. control. (**I**) Representative images of tetramethylrhodamine ethyl ester (TMRE)-stained adult cardiomyocytes isolated from hearts of the indicated genotype of mice at 8 weeks of age. Scale bar is 50 µm. (**J**) Representative heart images of transmission electron microscope (TEM) at 8 weeks of age from the indicated genotypes of mice. White arrows indicate mitophagy. Scale bars are shown in the figure panels. Data are scatter plot and bar with mean ± SEM (**F**–**H**).

To investigate the role that Mic60 protein plays within the MICOS complex in organizing mitochondrial structure and function in the mouse heart, we crossed *Immt*-loxP (fl) targeted mice with transgenic mice expressing Cre recombinase from the Myh6 promoter that normally drives cardiac-specific expression of the α-myosin heavy chain protein (see Materials and Methods and Figure 1A). Hearts were taken from control (*Immt*^+/+*-Myh6-Cre*^) versus *Immt^fl^*^/*fl-Myh6-Cre*^ mice at 8 weeks of age to assess protein expression. The data show efficient deletion of the *Immt* gene by loss of Mic60 protein, as well as major reductions in Mic19, Sam50, Mic26, and Mic27 of the greater MICOS complex in hearts of *Immt^fl^*^/*fl-Myh6-Cre*^ mice versus control (Figure 1B). Moreover, immunohistochemistry from these same hearts showed an almost complete loss of Mic19 protein in mitochondria from *Immt^fl^*^/*fl-Myh6-Cre*^ mice versus control (Figure 1C). *Immt^fl^*^/*fl-Myh6-Cre*^ mice failed to survive beyond 12 weeks of age versus control lines, indicating that deletion of this gene results in early adulthood lethality (Figure 1D) with extreme cardiomyopathy (Figure 1E). Indeed, echocardiographic assessment of ventricular function in 8-week-old *Immt^fl^*^/*fl-Myh6-Cre*^ mice versus control showed a significant loss of cardiac systolic function (Figure 1F), as well as cardiac hypertrophy measured by assessment of ventricular weight to body weight, and pulmonary edema measured by lung weight normalized to body weight (Figure 1G,H). Isolation of adult cardiomyocytes at 8 weeks of age showed a strong reduction in TMRE fluorescence from hearts of *Immt^fl^*^/*fl-Myh6-Cre*^ mice versus control, suggesting a loss of mitochondrial function (Figure 1I). Similarly, transmission electron microscopy of hearts from these mice showed severely disrupted mitochondrial architecture from *Immt^fl^*^/*fl-Myh6-Cre*^ mice at 8 weeks of age, such as large areas with mitophagy, areas with necrotic mitochondria, areas with extreme IMM packing, and even large areas of sarcomeres completely devoid of mitochondria with only remnant debris (Figure 1J and Supplementary Figure S1A–D).

**Figure 2:**
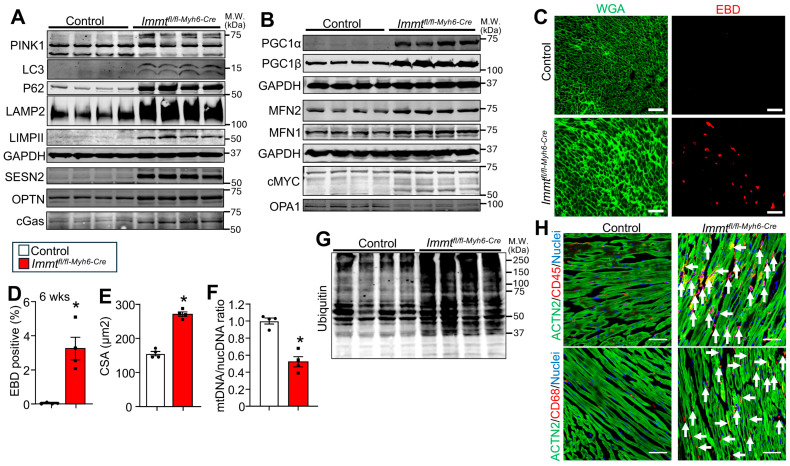
Characterization of heart-specific *Immt* gene-deleted mice. (**A**) Western blots of autophagy-associated proteins from hearts of *Immt^fl^*^/*fl-Myh6-Cre*^ mice versus control at 8 weeks of age. (**B**) Western blots of proteins involved in mitochondrial biogenesis and fusion from hearts of *Immt^fl^*^/*fl-Myh6-Cre*^ mice versus control at 8 weeks of age. Numbers indicate molecular weight (kDa). GAPDH is used as a loading control. (**C**) Representative heart histological images of Evans blue dye (EBD) and Alexa Fluor 488-conjugated wheat germ agglutinin (WGA) for membrane staining (green) in heart sections from *Immt^fl^*^/*fl-Myh6-Cre*^ mice versus control at 6 weeks of age. Scale bar is 100 µm. (**D**) Quantification of the EBD uptake; *n* = 4 in both groups. *: *p* = 0.0028 vs. control. (**E**) Quantification of cardiomyocyte cross-sectional area (CSA) in hearts of *Immt^fl^*^/*fl-Myh6-Cre*^ mice versus control at 8 weeks of age; *n* = 4 in both groups. *: *p* < 0.0001 vs. control. (**F**) Quantification of mitochondrial DNA (mtDNA)/nuclear DNA (nucDNA) ratio in hearts of control versus *Immt^fl^*^/*fl-Myh6-Cre*^ mice at 8 weeks of age; *n* = 4 in both groups. *: *p* < 0.0005 vs. control. (**G**) Western blot of ubiquitinated proteins from hearts of *Immt^fl^*^/*fl-Myh6-Cre*^ mice versus control at 8 weeks of age. Numbers indicate molecular weight (kDa). (**H**) Representative heart immunohistological images from *Immt^fl^*^/*fl-Myh6-Cre*^ mice versus control at 8 weeks of age, stained for sarcomeres in green (ACTN2), CD45 or CD68 inflammatory cells in red, or nuclei in blue. The white arrows show the position of CD45 or CD68 cells. Scale bars are 50 µm. Data are scatter plot and bar with mean ± SEM (**D**–**F**).

Consistent with this phenotype of lost or destroyed mitochondrial remnants, Western blotting for markers of mitophagy such as PTEN-induced kinase 1 (PINK1), microtubule-associated proteins 1A/1B light chain 3B (LC3), P62 (SQSTM1), SESN2 (Sestrin2), OPTN (Optineurin) and cGas were highly upregulated in the hearts of *Immt^fl^*^/*fl-Myh6-Cre*^ mice versus control mice (Figure 2A). Western blotting also showed a major expansion in lysosomes as measured by expression of lysosome-associated membrane protein 2 (LAMP2) and lysosomal integral membrane protein II (LIMPII, also called SCARB2) in the hearts of *Immt^fl^*^/*fl-Myh6-Cre*^ mice versus control mice (Figure 2A). Finally, Western blotting for the known effector transcriptional regulators of mitochondrial biogenesis such as peroxisome proliferator-activated receptor gamma, coactivator 1 alpha (PGC1α) and peroxisome proliferator-activated receptor gamma, coactivator 1 beta (PGC1β), and cMYC were dramatically upregulated in the hearts of *Immt^fl^*^/*fl-Myh6-Cre*^ mice versus control, while expression of mitofusin 1 (MFN1) and mitofusin 2 (MFN2) were upregulated by 2-fold yet OPA1 (Optic Atrophy 1) was downregulated in *Immt* deleted hearts (Figure 2B). These data indicate that hearts from *Immt^fl^*^/*fl-Myh6-Cre*^ mice are undergoing programs of mitophagy and mitobiogenesis simultaneously in an attempt to deal with extreme disruption and loss of cardiac mitochondria and loss of metabolic function.

We also quantified cardiomyocyte necrosis over a 24 h period in the hearts of *Immt^fl^*^/*fl-Myh6-Cre*^ mice versus control mice at 6 weeks of age using i.p. injection of Evans blue dye (EBD) and subsequent fluorescence analysis of histological sections the next day, with co-staining of membranes with Alexa Fluor 488-conjugated wheat germ agglutinin (Figure 2C). Quantitation of the data showed almost 4% of cardiomyocytes were EBD-positive over 24 h in the hearts of *Immt^fl^*^/*fl-Myh6-Cre*^ mice at 6 weeks of age versus control mice (Figure 2D). By 8 weeks of age, individual myocytes showed significantly larger cross-sectional area and hypertrophy, as well as a 2-fold loss of total mitochondrial DNA content, reflecting a loss in the biogenesis process to ongoing mitophagy due to loss of the MICOS complex (Figure 2E,F). Indeed, total protein isolation from the hearts of *Immt^fl^*^/*fl-Myh6-Cre*^ mice at 8 weeks of age showed large increases in total ubiquitinated proteins (Figure 2G). Heart sections were also evaluated from *Immt^fl^*^/*fl-Myh6-Cre*^ mice versus control mice at 8 weeks for immunohistochemistry for total inflammatory leukocytes with CD45 antibody or activated macrophages with CD68 antibody, and the data showed noticeably increased inflammation in the hearts of the *Immt* gene-deleted mice (Figure 2H).

**Figure 3:**
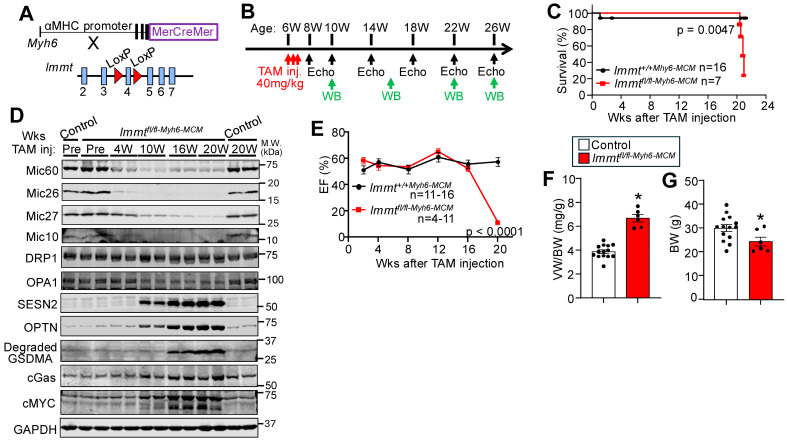
Generation and analysis of heart-specific and adult deletion of *Immt*. (**A**) To generate inducible cardiomyocyte-specific *Immt* knockout (*Immt^fl^*^/*fl-Myh6-MCM*^) mice, loxP site-targeted *Immt* mice were crossed with αMHC promoter (Myh6)-driven MerCreMer transgenic mice. (**B**) Experimental timeline on *Immt^fl^*^/*fl-Myh6-MCM*^ mice and controls. To delete *Immt* locus, 40 mg/kg body weight of tamoxifen (TAM) was injected intraperitoneally 3 times every other day and the mice were analyzed over the time line given by echocardiography or Western blotting. (**C**) Survival rate after TAM injection in the indicated genotypes of mice. (**D**) Protein levels of Mic60, Mic26, Mic27, Mic10, DRP1 and OPA1 mitochondrial proteins in hearts of the 2 groups of mice at each of the different time points shown after tamoxifen administration (TAM). Also shown are autophagy and mitophagy proteins SESN2, OPTN, degraded GSDMA, cGas, and the mitochondrial biogenesis gene cMYC. Glyceraldehyde 3-phosphate dehydrogenase (GAPDH) is used as a loading control. (**E**) Time course of ejection fraction (EF) measured by echocardiography in the 2 genotypes of mice shown. (**F**) Ventricular weight/body weight ratio (VW/BW) and (**G**) body weight (BW) at 20 weeks after TAM injection in control and *Immt^fl^*^/*fl-Myh6-MCM*^ mice; *n* = 14 in control and *n* = 6 in *Immt^fl^*^/*fl-Myh6-MCM*^ mice. *: *p* < 0.001 vs. control. Data are scatter plot and bar with mean ± SEM (**F**,**G**).

We also performed an entirely independent approach in adult *Immt^fl^*^/*fl*^ mice crossed into the Myh6-MerCreMer (Myh6-MCM) transgenic background, which permits inducible and heart-specific gene deletion with tamoxifen administration (see Materials and Methods, Figure 3A). Control (*Immt*^+/+*-Myh6-MCM*^) and *Immt^fl^*^/*fl-Myh6-MCM*^ mice were given tamoxifen at 6 weeks of age, three times over six days, then subjected to echocardiography at 8, 10, 14, 18, 22 and 26 weeks of age (Figure 3B). Approximately 20–22 weeks after tamoxifen administration, the *Immt^fl^*^/*fl-Myh6-MCM*^ mice succumbed to lethality (Figure 3C). Western blotting from the hearts of these *Immt^fl^*^/*fl-Myh6-MCM*^ mice and controls showed that Mic60 protein, as well as Mic26, Mic27, and Mic10 began to disappear by 4 weeks after tamoxifen injection, and then fully depleted by 10 weeks and beyond (Figure 3D), as these mice began to progressively lose cardiac function as measured by echocardiography (Figure 3E) and show increases in cardiac hypertrophy (Figure 3F). These mice also lost body weight, given the severe disruption in heart function and metabolic crisis, as also observed in heart failure patients with cachexia (Figure 3G). Consistent with this phenotype, Western blotting from *Immt* targeted hearts also showed induction of autophagy and mitophagy proteins (SESN2, OPTN, and GSDMA) as shown in Figure 2A,B by 10 weeks after tamoxifen administration, as well as induction of cGas and cMYC versus control hearts before a reduction in cardiac function was observed (Figure 3D,E). We also observed a reduction in OPA1 protein at 10, 16, and 20 weeks after tamoxifen administration, but no change in DRP1 (Figure 3D). Thus, adult deletion of *Immt* in the heart also results in lethality due to a progressive loss of mitochondrial function in mice, similar to lethality observed within 2 weeks after tamoxifen administration in *Immt*-loxP targeted using the ubiquitous *Rosa26*-CreERT2 allele to delete Mic60 protein from all tissues and cells in vivo [14]. However, the induction of the mitochondrial stress response with autophagy and mitochondrial biogenesis began as soon as the MICOS complex of proteins was lost 4 to 10 weeks after tamoxifen induction, but considerably before cardiac function was negatively impacted (Figure 3D).

### 3.2. Immt Heart-Specific Null Mice Show Mitochondrial Stress Response

**Figure 4:**
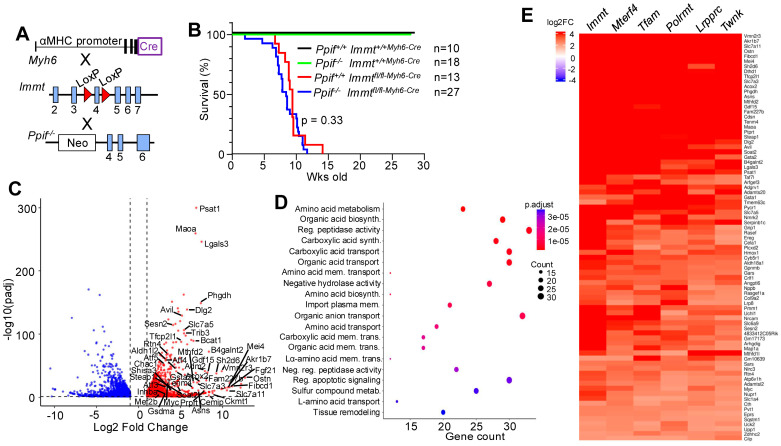
Phenotypes of heart-specific *Immt* gene-deleted mice. (**A**) To generate *Immt* and *Ppif* double gene deletion, *Mic60^fl^*^/*fl-myh6-Cre*^ mice were crossed with *Ppif* gene-deleted mice. (**B**) Survival rate of *Ppif*^+/+^ *Immt*^+/+*-Myh6-Cre*^, *Ppif^−/−^ Immt*^+/+*-Myh6-Cre*^*, Ppif*^+/+^ *Immt^fl^*^/*fl-myh6-Cre*^, *Ppif*^−/−^ *Immt^fl^*^/*fl-Myh6-Cre*^ mice after birth. (**C**) Volcano plot of bulk RNA-seq comparing heart gene expression in *Immt*^+/+*-Myh6-MCM*^ and *Immt^fl^*^/*fl-Myh6-MCM*^ mice. Mitochondrial stress response gene names are also indicated. The red dots show selected genes that are significantly increased in expression while the blue dots show genes that are significantly decreased in mRNA expression. (**D**) Top 20 enriched GO-biological process terms with upregulated genes in *Immt^fl^*^/*fl-Myh6-MCM*^ mouse hearts compared to *Immt*^+/+*-Myh6-MCM*^ control hearts. (**E**) Heat map of universal mitochondria stress response genes (81 genes) identified by cross-referencing hearts of cardiomyocyte-specific *Immt*, *Twnk*, *Tfam*, *Lrpprc*, *Mterf4* and *Polrmt* gene-deleted mice.

We have previously shown that deletion of the *Ppif* gene encoding cyclophilin D (CypD) in the heart or skeletal muscle results in reduced myocyte necrosis from stimuli that otherwise cause calcium overload-induced cell death, such as muscular dystrophy or ischemia–reperfusion injury [23,35]. Hence, we crossed mice with the *Immt* gene deletion with the *Ppif* null mice to determine if the mechanism of cellular necrosis in the heart was due to mitochondrial calcium overload (Figure 4A) [36]. However, the data showed no protection by deletion of the *Ppif* gene in the hearts of *Immt^fl^*^/*fl-Myh6-Cre*^ mice, as these mice still showed early lethality (Figure 4B). These results suggest that the loss of the MICOS complex from mitochondria in the heart results in a form of myocyte death that is independent of calcium overload, further suggesting that Mic60 is not a direct regulator of cyclophilin D (*Ppif* gene product) as previously suggested [36]. To investigate the underlying mechanisms of death in *Immt^fl^*^/*fl-Myh6-MCM*^ mice, or if viewed another way, the mechanisms of compensation that allow these mice to live for up to 20 weeks after loss of mitochondrial function, we first performed RNA-seq analysis in hearts of *Immt^fl^*^/*fl-Myh6-MCM*^ mice at 20 weeks after tamoxifen injection when these hearts are in failure versus control. The results showed a remarkable profile of altered gene expression, with some genes induced more than 5000-fold (Figure 4C). Three general classes of genes represented the most significantly changed and most highly upregulated including, (1) secreted factors generated by the heart to signal the metabolic catastrophe that was unfolding, including *Ostn*, *Bmp10*, *Fibcd1*, *Fgf21*, *Gdf15*, *Adm2*, *Inhba*, *Retnlg*, *Vgf*, *Cxcl13* and *Lep*, (2) transcriptional regulators underlying metabolism, mitophagy and mitobiogenesis, including *Tfcp2l1*, *Myc*, *Atf4*, *Atf3*, *Atf5*, *Mef2b* and *Nrip3*, and (3) metabolic regulators that attempt to compensate for lost oxidative phosphorylation including *Fam227b*, *Past1*, *Maoa*, *Bcat1*, *Mthfd2*, *Trib3*, *Avil*, *Sesn2*, *Rtn4*, *Gsdma*, *Adrb3, Acox2,* and *Ckmt1* (Figure 4C,D). Supplementary Figure S2 gives the raw RNA-seq counts and statistics for many of these and other major changes in gene expression due to loss of *Immt* in the heart, as well as a summary of gene function. These results show a remarkable gene expression profile of stress in which genes that control mitochondrial clearance and biogenesis are simultaneously induced, as well as induction of genes that would compensate for lost fatty acid oxidation and mitochondrial energy production.

The mitochondrial stress response uncovered in the hearts of *Immt^fl^*^/*fl-Myh6-MCM*^ mice was very similar to the published RNA-seq profile in mice in which five critical mitochondrial-specific genes were separately deleted in the heart using a similar cardiac-specific Cre expressing approach, and these mice were similarly examined at a more terminal time point with altered cardiac function [21]. These mice had loxP targeting of *Tfam, Twnk, Lrpprc, Polrmt*, or *Mterf4* to generate cardiac-specific nulls, each of which also succumbed to early lethality. These five previous data sets were compared with the RNA-seq data from the *Immt^fl^*^/*fl-Myh6-MCM*^ mice, showing a highly similar profile of the same three overall classes of compensatory upregulated genes (Figure 4E). We also observed a reduction in expression of several genes involved in fatty acid oxidation, cellular adhesion, and membrane signaling with cardiac-specific deletion of the *Immt* gene (see Supplementary Figure S2 and GSE312894). Finally, of the 81 genes that were selected as commonly induced at part of the mitochondrial stress response (Figure 4E), we also generated RNA from the hearts of *Immt^fl^*^/*fl-Myh6-MCM*^ mice after 10 weeks of tamoxifen before overt pathology and lost function was observed, which showed induction of 57 of the 81 genes, which likely represent the immediate early mitochondrial stress response genes, not directly influenced by cardiomyopathy (Supplementary Figure S3). These data collectively suggest that loss of mitochondrial function in the heart invokes a universal stress response and a gene expression signature that attempts to compensate for this lost metabolic capacity, with a more immediate and primary set of genes becoming expressed at the very onset of mitochondrial dysfunction before there is cardiomyopathy.

### 3.3. Metabolic Changes in Hearts of Immt^fl/fl-Myh6-Cre^ Mice

**Figure 5:**
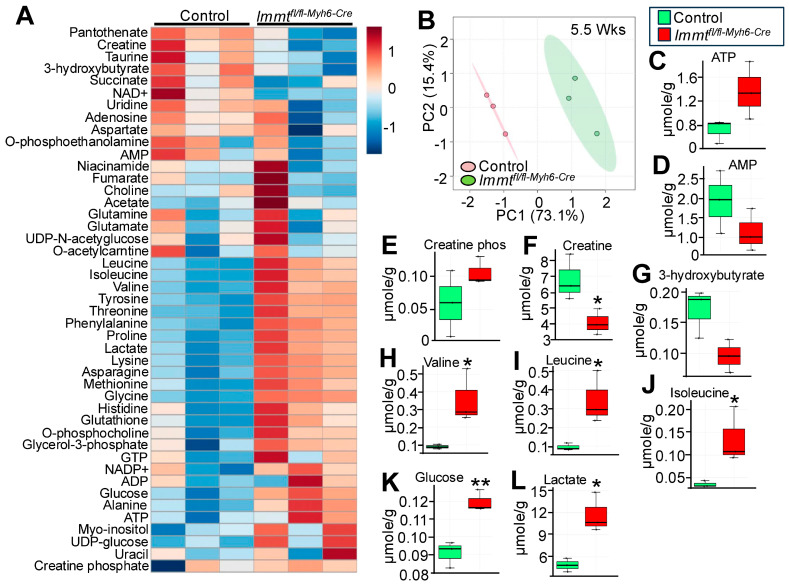
Metabolome analysis of *Immt^fl^*^/*fl-myh6-Cre*^ hearts at 5.5 weeks of age. (**A**) Heat map of 45 metabolites quantified in all 6 samples, including 3 *Mic60*^+/+*-Myh6-Cre*^ hearts (Control) and 3 *Mic60^fl^*^/*fl-Myh6-Cre*^ hearts. (**B**) Principal component analysis of the 2 genotypes of mice and the metabolic signature. (**C**,**D**) ATP (**C**) and AMP (**D**) levels. (**E**,**F**) Creatine phosphate (**E**) and creatine (**F**) levels. *: *p* < 0.05 vs. control. (**G**) The 3-hydroxybutyrate levels. (**H**–**J**) Valine (**H**), leucine (**I**), and isoleucine (**J**) levels. *: *p* < 0.05 vs. control. (**K**,**L**) Glucose (**K**) and lactate (**L**) levels. *: *p* < 0.05 vs. control. **: *p* < 0.01 vs. control; *n* = 3 in both groups. Data are shown as box and whisker plots with median line and scatter plot (**C**–**L**).

The bulk RNA-seq data suggested a gene expression profile whereby the heart attempts to use glucose as a source of energy, increase activity of the 1-carbon metabolic pathway [37], increase influx of amino acids and ketones [38] for greater reducing capacity. To more directly measure metabolites, hearts were taken from control mice or *Immt^fl^*^/*fl-**Myh6**-Cre*^ mice at 5.5 weeks of age for metabolomics analysis (Figure 5A,B). Hearts with *Immt* deletion showed a major increase in glucose, UDP-glucose, glycerol-3-phosphate, O-phosphocholine, glutathione, lactic acid, and most amino acids, including valine, leucine, isoleucine, tyrosine, threonine, phenylalanine, proline, lysine, asparagine, methionine, glycine, alanine and histidine (Figure 5A,H–K). AMP, creatine, 3-hydroxybutyrate, NAD+, succinate, adenosine, and uridine levels were reduced, yet ATP levels were increased (Figure 5A,C–G). The large increase in lactate in the hearts of *Immt* deleted mice also suggests a strong reliance on glycolysis (Figure 5L). Collectively, this profile suggests a shift in overall cardiac metabolism towards glucose utilization and amino acid shunts with augmented 1-carbon pathway function to maintain reductive potential and homeostatic balance for both catabolism and anabolism. Indeed, analysis of compensated gene expression and altered metabolic pathways in hearts of the *Twnk*, *Tfam*, *Lrpprc*, *Mterf4* and *Polrmt* gene-targeted mice discussed above also showed major shifts away from mitochondrial OXPHOS activity and towards glycolysis with increased glutathione redox reactions, a profile that was also associated with increased *Atf4* and *Myc* transcriptional activity [21], consistent with the observed large increase in cMYC protein in the heart (Figure 2B and Figure 3D).

## 4. Discussion

To investigate the importance of the MICOS complex in vivo, we used a mouse model with conditional and cardiac-specific deletion of the *Immt* gene, which encodes the Mic60 protein. A recent publication used a similarly designed conditional allele of *Immt* to delete the Mic60 protein in the adult mouse using a ubiquitous, but conditional CreERT2 fusion protein driven by the *Rosa26* locus, which resulted in lethality approximately 12 days later with widespread defects in many tissues due to loss of mitochondrial function [14]. Other approaches resulting in knockdown or loss of the Mic60 protein in yeast through mammalian cells have also suggested a necessary function for this protein in maintaining the MICOS complex and the proper formation of CJs and IMM stacking versus positioning of the OMM and its attachment to other structures [39,40]. The MICOS complex and the organization of the CJs is also required for the proper organization of the OXPHOS complexes and associated exchanger proteins that allow for efficient ATP generation through metabolic process resulting in fatty acid and glucose oxidation, but also usage of ketones, lactic acid and amino acids through the tricarboxylic acid (TCA) cycle, which also generates NADH and FADH_2_ for carrying electrons and generating reducing potential [3,41,42,43]. Thus, the MICOS complex and Mic60 are critical regulators of mitochondrial function, and disruption of this organizing principle is catastrophic for this organelle, resulting in lost OXPHOS function and the negative mitochondrial membrane potential that drives F_1_F_O_-ATPase activity, secondarily resulting in mitochondrial rupture, necrosis, and mitophagy.

It was interesting that loss of the *Immt* gene specifically from the heart did not result in a more immediate lethal event, especially given the extreme energy requirement of this organ and the fact that mitochondria occupy up to 1/3rd of the volume of an adult cardiomyocyte. By comparison, deletion of the *Immt* gene throughout the mouse in adulthood using the *Rosa26*-CreERT2 allele produced more immediate lethality in 12 days, which is actually much faster given the need for Mic60 protein turnover and the known long half-life of IMM proteins [14]. We observed efficient deletion of the Mic60 protein in the hearts of adult *Immt^fl^*^/*fl-Myh6-MCM*^ mice after 4 weeks of tamoxifen administration, yet these mice were viable up to 16–18 additional weeks despite a near-complete loss of OXPHOS capacity. We believe this protracted viability is due to compensation through the induction of the mitochondrial stress response that resulted in very dramatic changes in gene expression, likely resulting in the ability of the heart to generate ATP through induction of glycolysis in conjunction with induction of other shunt pathways that have yet to be fully identified. These metabolites and the secreted factors produced by the heart would promote glucose uptake and usage from circulation, like how tumors in advanced cancer can take over total body metabolism, resulting in extreme cachexia [44]. Indeed, the mitochondrial stress response gene signature observed in the hearts of the *Immt^fl^*^/*fl-Myh6-MCM*^ mice led to a significant loss of body weight, suggesting changes in total body metabolism and breakdown of other tissues to send glucose and metabolites into circulation for the heart to use (maybe first processed in the liver). Genes encoding such secreted factors included *Ostn*, *Bmp10*, *Fibcd1*, *Fgf21*, *Gdf15*, *Adm2*, *Inhba*, *Retnlg*, *Vgf*, *Cxcl13* and *Lep*, each of which can dominantly impact systemic metabolic function in some manner [45,46]. For example, secretion of either CXCL13 or ADM2 can promote thermogenesis in mice and greater caloric usage [47,48]. FGF21 and GDF15 are also known to be secreted in response to deficiencies in mitochondrial function, which then signals outwards to impact total body metabolism [49,50], which is also consistent with the total reduction in body weight observed in the *Immt^fl^*^/*fl-Myh6-MCM*^ mice 20 weeks later.

As stated above, mice with cardiac-specific deletion of necessary mitochondrial genes such as *Twnk*, *Tfam*, *Lrpprc*, *Mterf4* and *Polrmt* resulted in loss of OXPHOS, mitochondrial dysgenesis and rupture in the heart [21], producing a similar protracted lethal phenotype as observed in our heart-specific *Immt* deleted mice. These five other gene-deleted mouse models showed a very similar mitochondrial stress response gene signature, suggesting that this is a universal attempt at compensation when the heart loses mitochondrial function [21]. These five mouse models also showed induction of the transcription factors *Atf4*, *Atf5* and *Myc*, along with induction of genes underlying mitochondrial biogenesis, mitophagy, and induction of shunt and one-carbon metabolic pathways, such as upregulation of *Mthfd2*. However, loss of the Mic60 protein uniquely resulted in the induction of *Bmp10*, *Ckmt2*, *Cemip*, *Fpr1*, *Mlana*, and *Cxcl13*, which was not observed in the five other heart-specific KO mouse models [21]. These results collectively underscore the importance of Mic60 and the MICOS complex in supporting mitochondria structure-function, and that without this complex, mitochondria degenerate and OXPHOS activity is lost. The unexpected finding is that the mouse heart was able to temporarily compensate for the loss of mitochondria in supplying high-energy phosphate for contraction and ion handling, suggesting clues underlying the metabolic resilience of this organ mediated by the mitochondrial stress response gene signature that was observed and potential novel shunt pathways, or even that partial free-floating IMM fragments without the OMM can still mediate some degree of oxidative phosphorylation of limited substrates at one or more of the complexes.

## 5. Conclusions

Our results show that the *Immt* gene is required in the mouse heart for maintaining normal mitochondrial function and OXPHOS activity, and that the *Immt* gene product directly supports the stability of the MICOS complex and the organization of CJs and IMM stacking in vivo. Loss of the Mic60 protein in the mouse heart led to lethality due to loss of mitochondrial OXPHOS function, although lethality occurred in a protracted time frame due to extreme compensatory measures whereby the heart induced expression of mitochondrial stress response genes enabled partial metabolic activity and effective clearance of mitochondrial debris.

## Data Availability

The data presented in this manuscript are openly available. The raw RNA-seq expression data were submitted to the Gene Expression Omnibus (GEO) at https://www.ncbi.nlm.nih.gov/geo (accessed on 2 March 2026) with an accession number of GSE312894 and GSE322575. All other data are contained within the manuscript or Supplementary Materials.

## Supplementary Materials

The following supporting information can be downloaded at: https://www.mdpi.com/article/10.3390/cells15060505/s1, Figure S1: Transmission EM of heart images from *Immt^fl^*^/*fl-Myh6-Cre*^ mice at 8 weeks of age. (A) Region of tissue with widespread mitochondrial necrosis and mitophagy with irregular structures as shown in Figure 1. (B) Some regions of heart tissue show complete loss of mitochondria as noted with white arrowheads. (C) Some areas of heart tissue show areas of mitochondrial inner membranes that form blind parallel sacs or unconnected circles shown by white arrows. (D) Some areas of heart tissue show areas of IMM that from massive, repeated stacks with no lumen, as shown by yellow arrows. The scale bars are shown at the bottom of each panel; Figure S2: Mitochondrial stress response. The most highly upregulated genes from hearts of the cardiac-specific *Immt* gene deleted mice (KO) versus wildtype mice (WT) at 20 weeks after tamoxifen injection. The figure shows the gene name, gene description, raw RNA counts of KO and WT samples, log_2_-fold change, *p* value, and a description of the role that the gene plays in the mitochondrial stress response or metabolism; Figure S3: Heat map of differential gene expression in the hearts of *Immt^fl^*^/*fl-Myh6-MCM*^ mice at 16 versus 26 weeks of age (10 versus 20 weeks after tamoxifen treatment). The same 81 genes shown in Figure 4E are shown again from the heart specific *Immt* gene deleted mice at 26 weeks of age that were in heart failure (Figure 3E), but compared against RNA sequencing from hearts of *Immt* heart-specific deleted mice at 16 weeks of age when the mice are still fully compensated with no reduction in cardiac function (Figure 3E). Approximately 57 of the 81 genes were induced in the hearts of mice at 16 weeks of age, while 24 genes were not yet detected. This suggests that the first 57 induced genes at the 16-week time point represent the immediate early mitochondrial stress response genes that preceded loss of cardiac function or overt pathology, but that the majority of the induced genes are the same and even the same relative level of total induction versus control hearts; File S1: Original Western blot full size images showing the bands used in each of the Western blots throughout the paper, so that the identity of the protein bands in question can be verified and the size also inferred.
