# Supplementary material for: Heart-Specific and Conditional Deletion of the Immt Gene Reveals Its Role in Regulating Mitochondrial Structure and Total Heart Metabolism
Source: Cells. 2026 Mar 12;15(6):505. doi: 10.3390/cells15060505 (PMC13025846; doi:10.3390/cells15060505)
Supplement: Supplementary file 1 [file cells-15-00505-s001.zip › SuppFigs.R2.FINAL.pdf]

Supplementary Figure S1

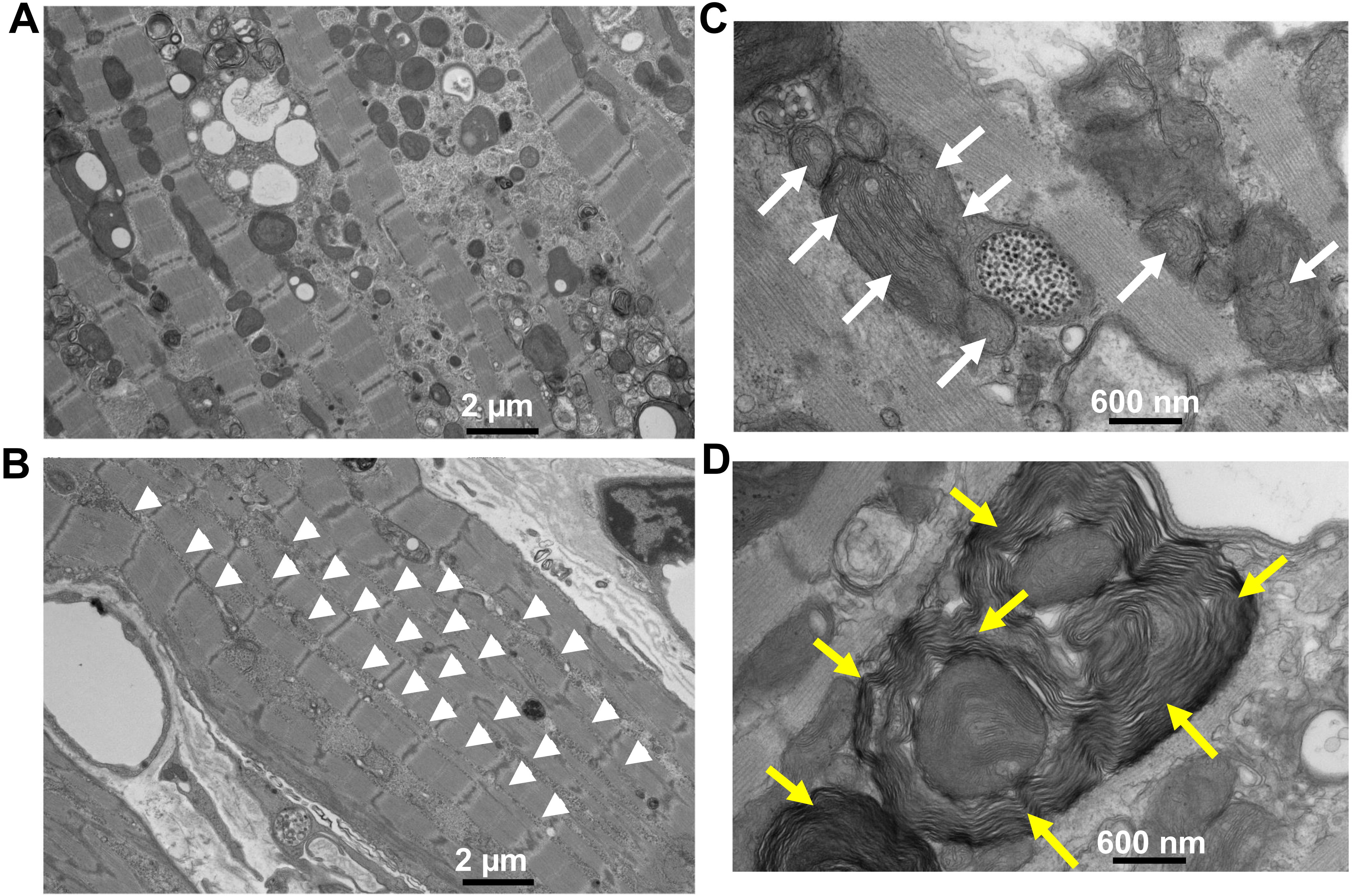

Supplementary Figure S1. Transmission EM of heart images from *Immt*$^{fl/fl\text{-}Myh6\text{-}Cre}$ mice at 8 weeks of age. (A) Region of tissue with widespread mitochondrial necrosis and mitophagy with irregular structures as shown in Figure 1. **(B)** Some regions of heart tissue show complete loss of mitochondria as noted with white arrowheads. **(C)** Some areas of heart tissue show areas of mitochondrial inner membranes that form blind parallel sacs or unconnected circles shown by white arrows. **(D)** Some areas of heart tissue show areas of IMM that from massive, repeated stacks with no lumen, as shown by yellow arrows. The scale bars are shown at the bottom of each panel.

Supplementary Figure S2.

Gene name	Gene Description	KO	WT	Log-2 Fold	P value	Role in mitochondrial biology or mito stress
Ostn	osteocrin	5099	0.77	12.66	7.30E-32	musclin enhances physical endurance by promoting mito biogenesis. KO mice have muscle energy defect, less mito
Fibcd1	fibrinogen C domain containing 1	495.5	0	11.16	2.55E-18	myokine that preserves myofiber size. Autosomal recessive muscular dystrophy in humans with homozygous mutation
Fgf21	fibroblast growth factor 21	395.9	0	10.83	5.79E-17	cytokine that can be induced by mitochondrial stress and to communicate mitochondrial stress with distant tissues
Mei4	meiotic double-stranded break formation protein 4	338.6	0	10.61	1.54E-16	loss of mei4 leads to defects in mitochondrial function and morphology, specifically in the context of cell division
Slc7a11	solute carrier family 7, member 11	3351	2.26	10.52	6.42E-12	cystine/glutamate antiporter for redox homeostasis and antioxidant defense by cystine uptake and glutathione synthesis
Sh2d6	SH2 domain containing 6	1203	1.99	9.257	1.44E-38	disrupting the SH2D6 gene leads to mito dysfunction, highlighting its importance in maintaining healthy mito function
Gdf15	growth differentiation factor 15	10660	36.5	8.188	6.82E-40	GDF15 distinguish patients with primary mito myopathy from other myopathies, including metabolic myopathies
Fam227b	family with sequence similarity 227, member B	520.7	1.9	8.055	1.43E-27	interacts with subunits of mito ATP synthases, which are crucial for energy production
Adm2	adrenomedullin 2	744.5	3.03	7.893	1.92E-42	expression is induced by various stressors, regulated by ATF4 and induced by mito respiration chain inhibition.
Ptprt	protein tyrosine phosphatase, receptor type, T	1182	8.54	7.109	9.28E-12	receptor-type protein tyrosine phosphatases (RPTPs), and its loss leads to changes in mitochondrial morphology and function
Slc7a3	solute carrier family 7, member 3	2353	18.6	6.977	3.29E-24	sodium-independent transport of cationic amino acids, which is induced when mitochondrial aerobic metabolism is inhibited.
Psat1	phosphoserine aminotransferase 1	13498	116	6.864	1.05E-303	involved in serine biosynthesis to regulate mito function and metabolism by production of α-ketoglutarate for glycolysis
Maoa	monoamine oxidase A	28665	262	6.775	4.19E-260	plays a role in regulating mito function and quality control, including processes like mito biogenesis, function, and mitophagy
Bcat1	branched chain aminotransferase 1, cytosolic	2173	23.3	6.536	4.60E-90	influences mitochondrial biogenesis and function, especially in the growth and proliferation of certain cancers.
Mthfd2	methylenetetrahydrofolate dehydrogenase 2	15655	169	6.534	7.14E-25	mito regulator of folate-mediated one-carbon metabolism. Requiered for DNA synthesis, repair, and purine biosynthesis
Asns	asparagine synthetase	16256	189	6.426	1.25E-15	ATF4 target and crucial role in mito function and metabolism as asparagine signals active respiration to ATF4
Dlg2	discs large MAGUK scaffold protein 2	7735	142	5.77	1.12E-139	research suggests a link between DLG2 and mitochondrial function and neurodevelopmental disorders
Trib3	tribbles pseudokinase 3	7976	187	5.413	8.05E-102	expression changed in response to mitochondrial respiratory chain inhibition. Trib3KO mice increased adiposity.
Avil	advillin	3336	92.8	5.168	4.29E-130	linked with mitochondrial function, including mitochondrial dynamics and oxidative stress
Tfcp2l1	transcription factor CP2-like 1	2293	68.1	5.074	1.57E-88	Tfcp2l1-induced fatty acid oxidation is vital for the survival of embryonic stem cells under metabolic stress. in nucleoli and mito
Shisa3	shisa family member 3	3432	185	4.215	1.34E-08	regulation of Wnt/beta-catenin signaling, expression in breast cancer cells leads to a reduced mito activity, tumor suppression
Sesn2	sestrin 2	3616	210	4.109	5.85E-115	mito quality control by localizing on the outer mito membrane and regulating mitophagy pathways
Chac1	ChaC, cation transport regulator 1	2303	185	3.633	3.13E-46	upregulated to promote the mobilization of cysteine from glutathione to support the synthesis of Fe-S clusters within mito
Inhba	inhibin beta-A	1883	159	3.566	4.34E-07	induces state IV oxygen consumption in mitochondria, and causes shift from glycolytic to oxidative phosphorylation
Myc	myelocytomatosis oncogene	1738	153	3.505	4.95E-24	a key regulator of cell growth and metabolism, in part by playing a crucial role in mitochondrial biogenesis and function.
Rtn4	reticulon 4	58429	5257	3.474	2.17E-69	RTN4, along with CLIMP-63, plays a role in regulating mito structure and function, independent of its role in ER structure
Gsdma	gasdermin A	98.11	8.79	3.466	1.34E-06	can disrupt both the inner and outer mito membranes, releasing mito proteins and DNA leading to pyroptotic cell death
Atf4	activating transcription factor 4	23447	2514	3.221	2.76E-59	promotes enhanced mito networking, protein handling, and the capacity for clearance. Regulates mito biogenesis
Aldh1l2	aldehyde dehydrogenase 1 family, member L2	5855	629	3.218	6.34E-61	It affects metabolites relevant to β-oxidation of fatty acids, with increased acylcarnitines and Krebs cycle intermediates
Atf3	activating transcription factor 3	1777	218	3.026	3.09E-17	ATF3 can both negatively and positively influence mitochondrial function depending on the cellular context.
Atf5	activating transcription factor 5	18935	2335	3.019	5.11E-52	regulates the mito unfolded protein response and expression of mito proteostasis genes, protects from mito stress

Supplementary Figure S2. Mitochondrial stress response. The most highly upregulated genes from hearts of the cardiac-specific *Immt* gene deleted mice (KO) versus wildtype mice (WT) at 20 weeks after tamoxifen injection. The figure shows the gene name, gene description, raw RNA counts of KO and WT samples, log2-fold change, P value, and a description of the role that the gene plays in the mitochondrial stress response or metabolism.

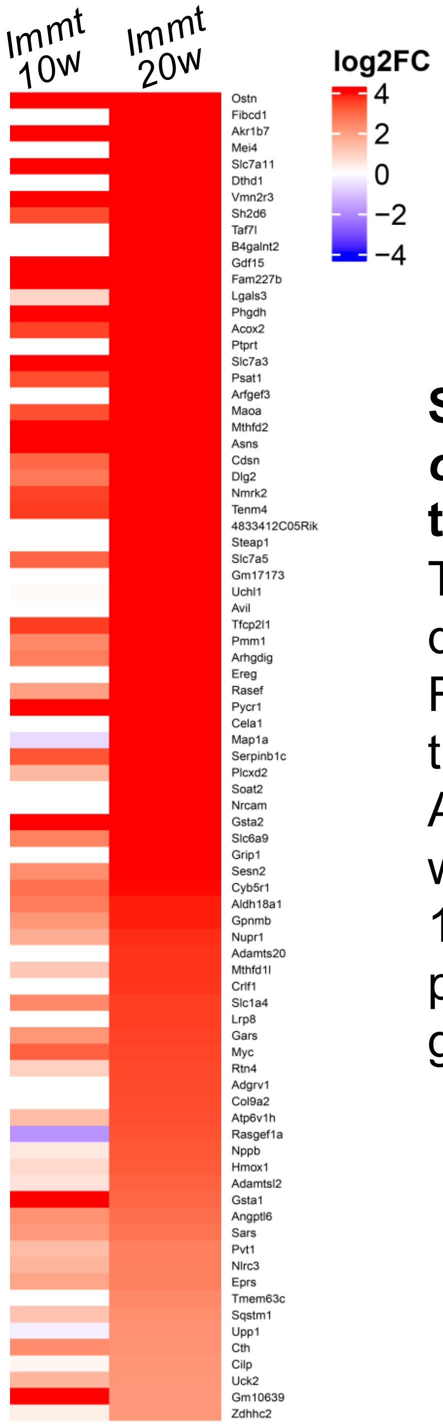

Supplementary Figure S3. Heat map of differential gene expression in the hearts *of Immt*$^{fl/fl\text{-}Myh6\text{-}MCM}$ mice at 16 versus 26 weeks of age (10 versus 20 weeks after tamoxifen treatment).

The same 81 genes shown in Figure 4E are shown again from the heart specific *Immt* gene deleted mice at 26 weeks of age that were in heart failure (Fig. 3E), but compared against RNA sequencing from hearts of *Immt* heart-specific deleted mice at 16 weeks of age when the mice are still fully compensated with no reduction in cardiac function (Fig. 3E). Approximately 57 of the 81 genes were induced in the hearts of mice at 16 weeks of age, while 24 genes were not yet detected. This suggests that the first 57 induced genes at the 16-week time point represent the immediate early mitochondrial stress response genes that preceded loss of cardiac function or overt pathology, but that the majority of the induced genes are the same and even the same relative level of total induction versus control hearts.
