# Supplementary figures and images for: Heart-Specific and Conditional Deletion of the Immt Gene Reveals Its Role in Regulating Mitochondrial Structure and Total Heart Metabolism
Source: Cells. 2026 Mar 12;15(6):505. doi: 10.3390/cells15060505 (PMC13025846; doi:10.3390/cells15060505)

Figure 1B

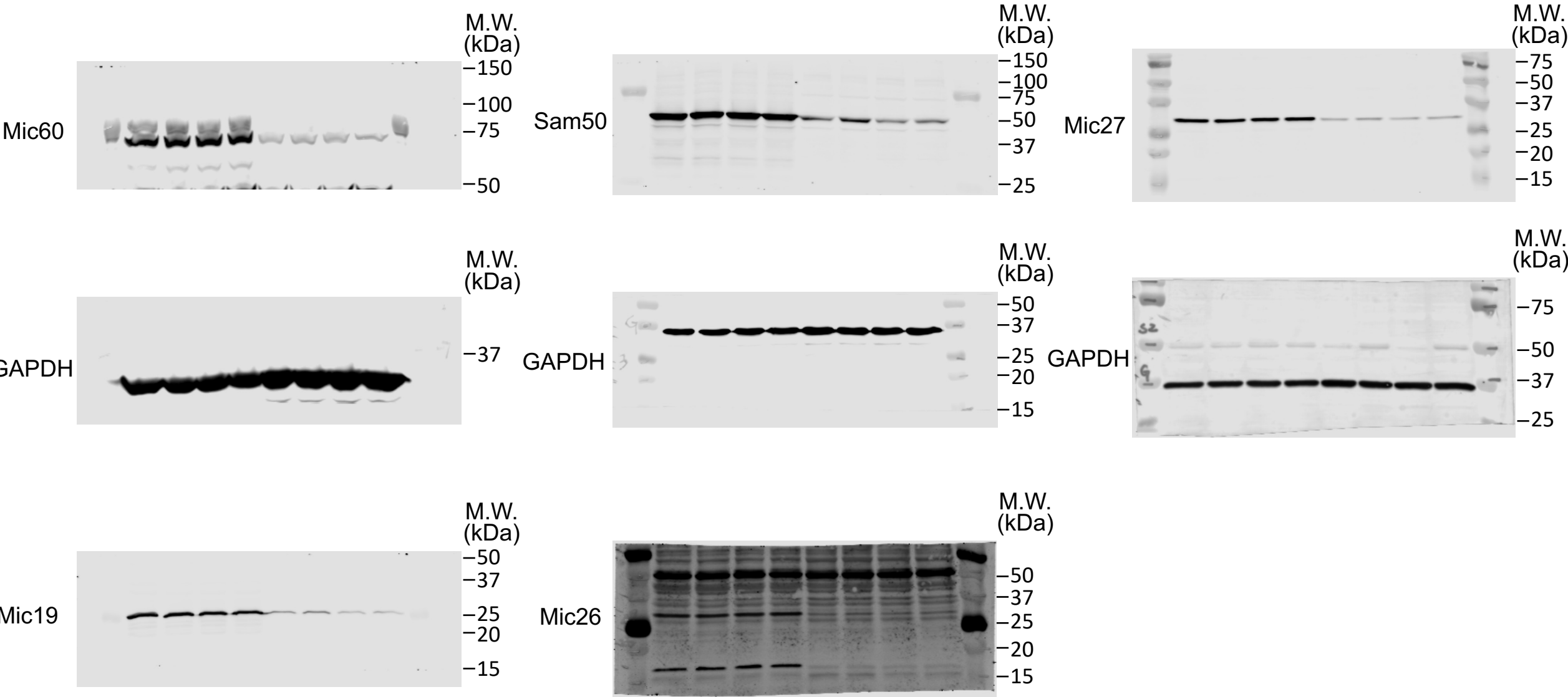

Figure 2A

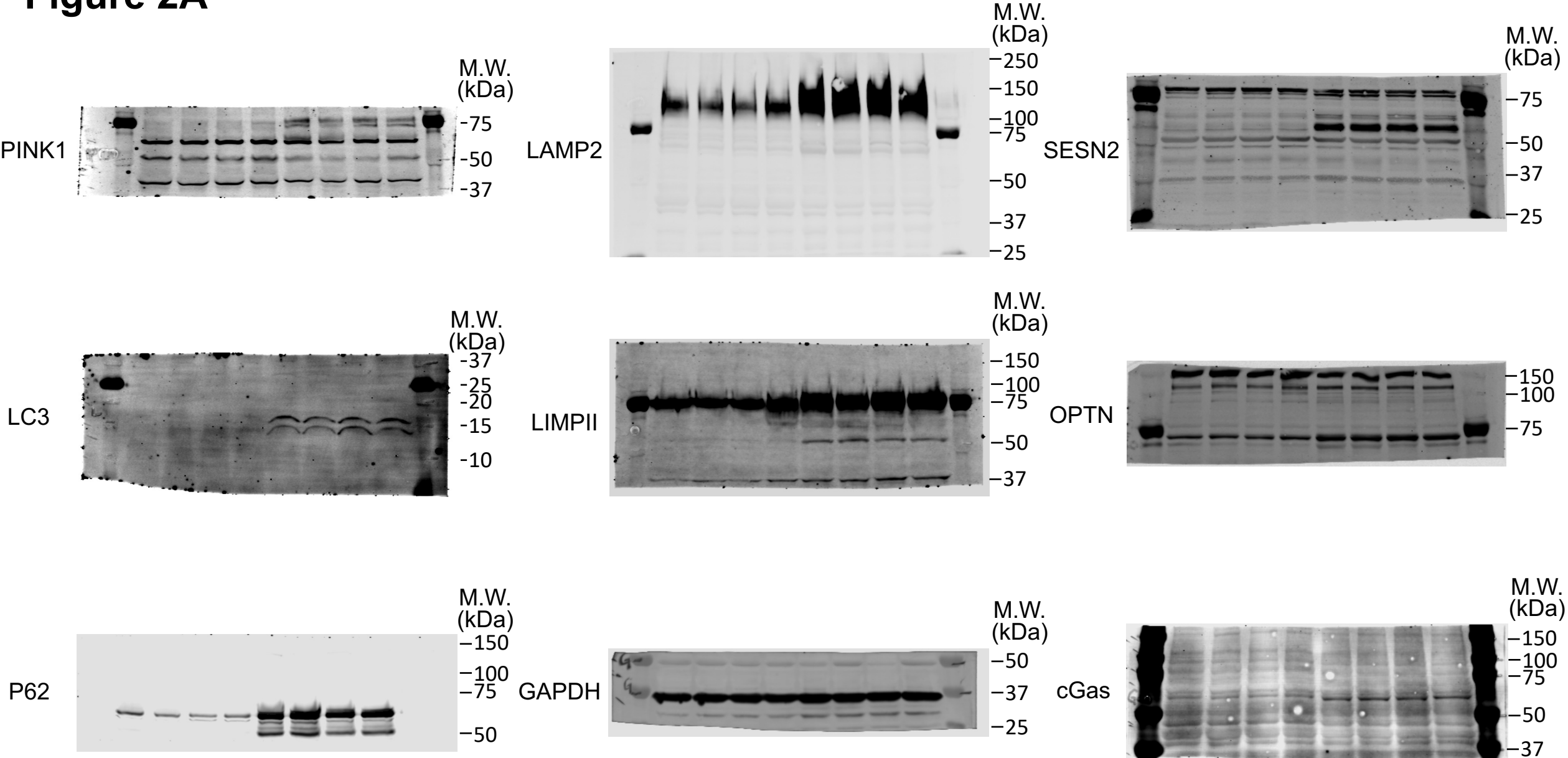

Figure 2B and 2G

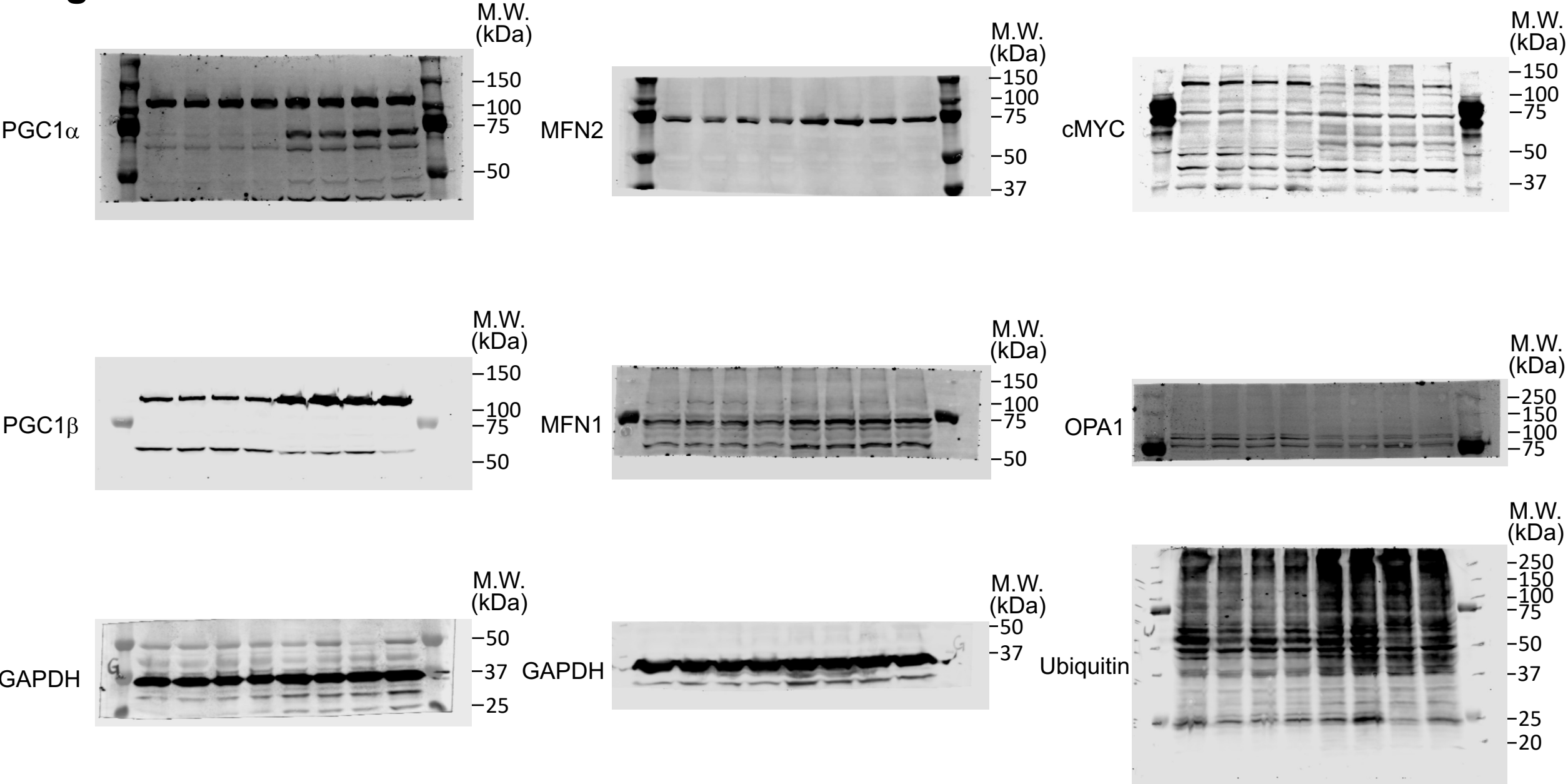

Figure 3D

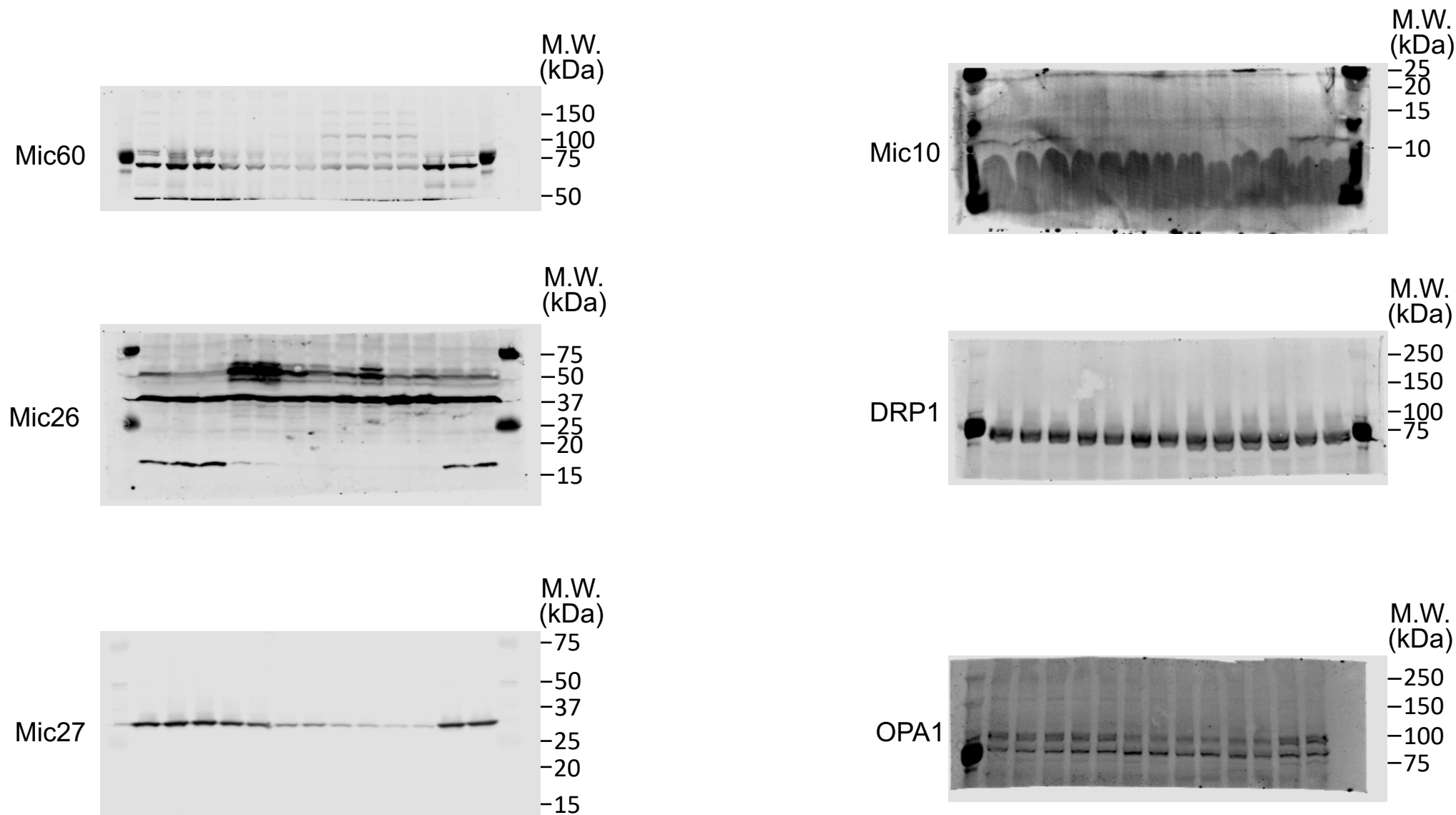

Figure 3D (continued)

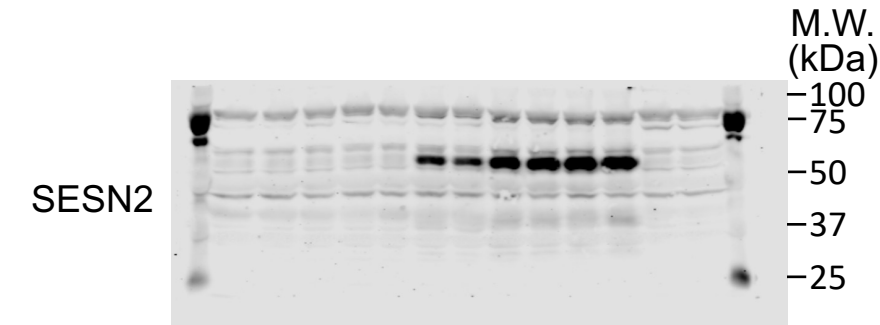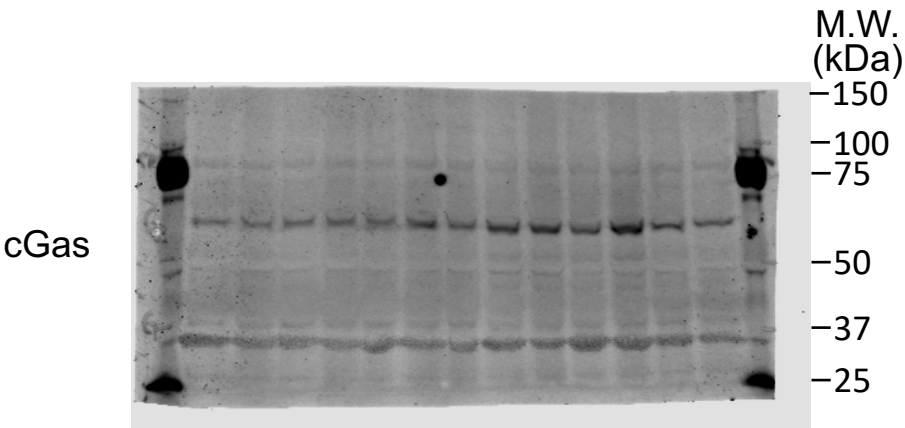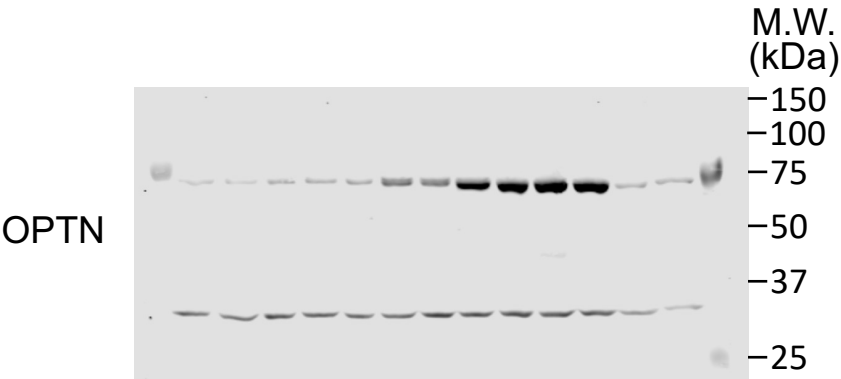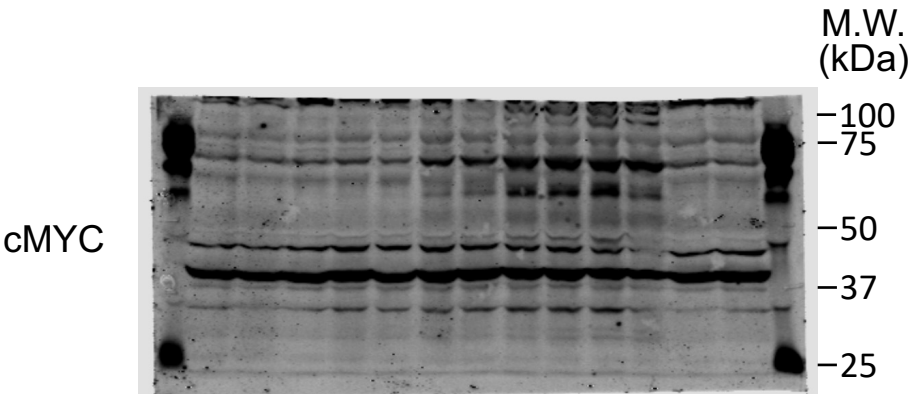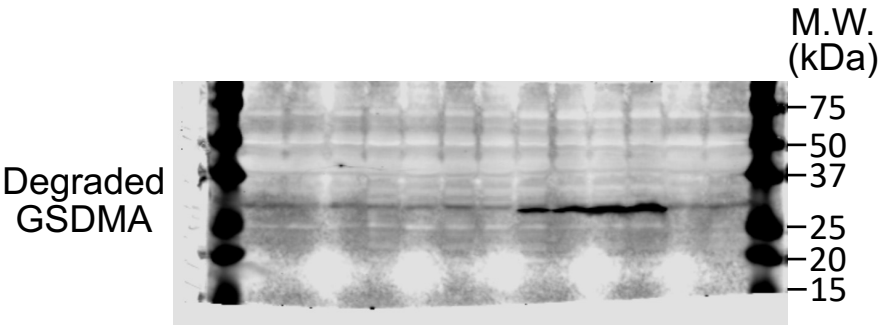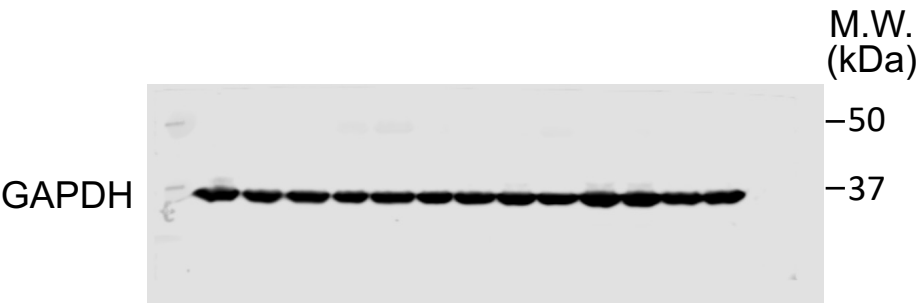

Supplement: Supplementary file 1 [file cells-15-00505-s001.zip › FileS1.OriginalBlots.R2.FINAL.pdf]
